# Effects of aerosolized citric acid–radio frequency as a pretreatment on hot‐air drying characteristics of banana

**DOI:** 10.1002/fsn3.2610

**Published:** 2021-09-28

**Authors:** Reza Ghorani, Mohammad Noshad, Behrooz Alizadeh Behbahani

**Affiliations:** ^1^ Department of Food Science and Technology Faculty of Animal Science and Food Technology Agricultural Sciences and Natural Resources University of Khuzestan Mollasani Iran

**Keywords:** aerosolized citric acid, banana, drying, radiofrequency

## Abstract

The effects of aerosolized citric acid–radio frequency (RF) pretreatment were evaluated on the quality characteristics of hot air‐dried banana. The results showed that increasing the RF intensity elevated the total phenolic content (TPC), shrinkage, and color changes, while the TPC and color changes decreased with increasing the RF exposure duration. A rise in the RF intensity reduced the rehydration ratio (RR) and firmness of the samples. Aerosolization of citric acid rendered the preservation of the phenolic compounds of the samples to a higher extent, and TPC decreased from 311 ± 3.4 mg/g in fresh banana to 252.1 ± 4.24 mg/g in the samples treated with a RF of 27.12 Hz for 40 min, 280.5 ± 8.1 mg/g in the ones treated with 1% aerosolized citric acid for 40 min, and 162.5 ± 10.8 mg/g in the ones with no pretreatment. According to scanning electron microscopy (*SEM*), the application of aerosolized citric acid pretreatment caused tissue softening and the formation of cell holes in the samples. Cell wall collapse and damage were severe when RF was in use, which caused the blockage of some microchannels within the tissue. The Page model with the highest determination coefficient (*R* ^2^) and the lowest root‐mean‐squared error (RMSE) and chi‐square (*χ* ^2^) was selected as the best model.

## Graphical abstract

- Aerosolized citric acid–radio frequency pretreatment was used as pretreatment for drying.
- Pretreatment of AC–RF improved the quality characteristics of the dried banana.
- The Page model was selected as the best model for drying kinetic.

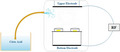

## 1 INTRODUCTION

Banana has high nutritional value due to its high content of potassium, vitamin C, starch, and sugar. However, like other fruits, it is perishable. Moisture is the predominant substance in most fruits and vegetables, which greatly impacts the susceptibility of food to spoilage by microorganisms or chemical reactions. Accordingly, a considerable amount of moisture must be eliminated from fruits and vegetables to control microbial spoilage and the adverse impacts of chemical reactions (Macedo et al., 2020; Takougnadi et al., 2020; Tunckal & Doymaz, 2020). Hot‐air drying (HAD) is the most common drying method, which includes the simultaneous transfer of mass and heat. The use of high temperatures or extended periods in traditional drying methods results in some undesirable physical and chemical changes in the product, such as changes in color, fragrance, and flavor in addition to nutrient loss, wrinkling, high energy consumption, and being a time‐consuming process (Forouzanfar et al., 2020; Noshad & Ghasemi, 2020; Roueita et al., 2020). Altogether, food quality could be preserved desirably by adopting other drying methods or a pretreatment, and the use of novel drying techniques that involve two or more pretreatments before drying has gained a great deal of attention. The adequate selection of drying methods may improve the quality of dried fruits and reduce the process time and energy consumption (Ashtiani et al., 2020; Li et al., 2021; Noshad & Ghasemi, 2020).

Browning is one of the most significant adverse changes that occurs in dried food products. It is caused by enzymatic and nonenzymatic reactions. Various acids such as citric acid, malic acid, and ascorbic acid are extensively used these days to reduce the enzymatic browning of dried products (Bonazzi & Dumoulin, 2011; Sarpong et al., 2018; Shah & Nath, 2008). Aerosolization is defined as the dispersion of a liquid or a solution in air in the form of fine mist (Oliveira et al., 2018). Therefore, instead of immersing the sample in the antibrowning solution, the aerosolization method can be used as a new antibrowning delivery technique.

Radio frequency (RF), with frequencies between 10 and 300 MHz, is a part of the electromagnetic spectrum. RF drying is an adequate means of rendering safe and high‐quality food products, as fast and consistent heating patterns, high penetration depth, and stable processing temperatures are the features of this method. Drying, thawing, disinfection, and pasteurization of food and agricultural products are some of the RF applications (Jiang et al., 2020; Wang et al., 2020; Zhou & Wang, 2019).

So far, no study has evaluated the simultaneous use of aerosolized citric acid and RF pretreatment in the HAD of banana. Therefore, in this study, the effects of aerosolized citric acid–RF pretreatment were investigated on the quality characteristics of the banana dried by HAD.

## 2 MATERIALS AND METHODS

Cavendish bananas with the same size and degree of ripening were bought from local market in Mollasani, Iran. After peeling, the bananas were cut into 10‐mm slices. The initial moisture content was determined using an oven at 95°C until reaching a constant weight.

### 2.1 Aerosolized citric acid under radio frequency exposure

**FIGURE 1:**
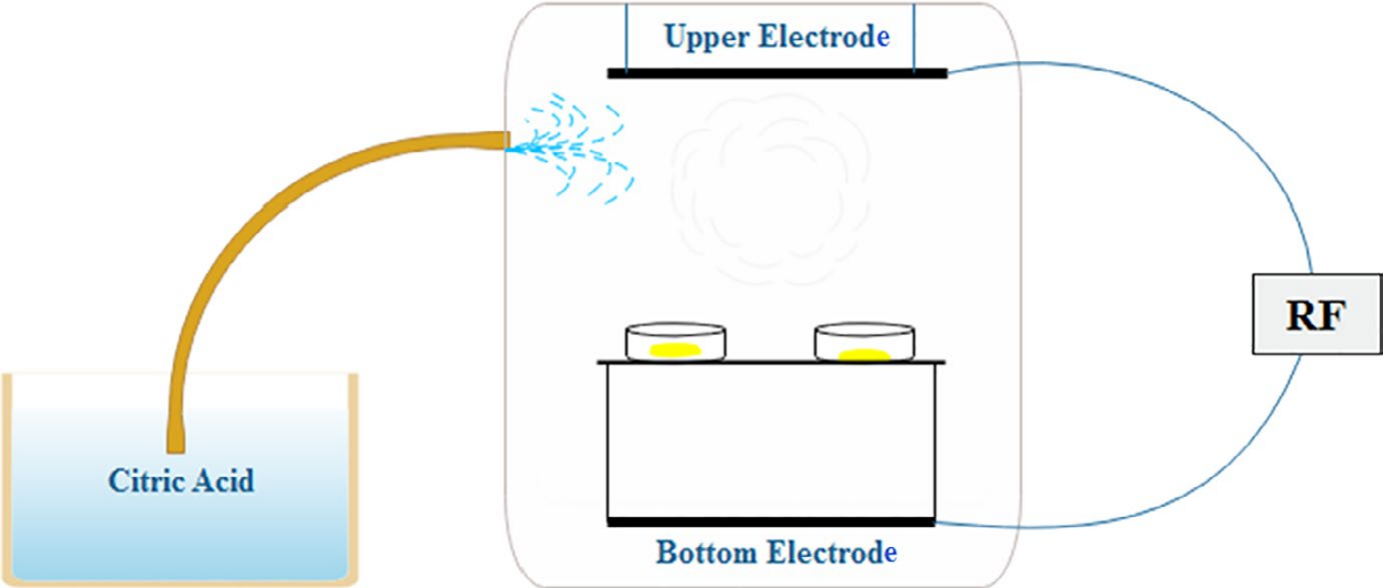
Set‐up used for aerosolized citric acid‐RF pretreatment before drying

The RF system was composed of parallel electrodes with a gap of 20 cm. Citric acid (1% v/v) was aerosolized (Figure 1). The simultaneous effects of aerosolized citric acid (1% v/v) and RF (13.56, 27.12, and 40 MHz for 40 and 80 min), as a pretreatment, were evaluated on the quality characteristics of the banana dried by HAD at 50°C.

### 2.2 Firmness

In order to evaluate the firmness of the samples, the penetration test was performed using a TA‐XT Plus texture analyzer (Stable Micro Systems) equipped with a 25‐N load cell and a cylindrical puncture probe, 6 mm in diameter, at a constant speed of 10 mm/min (Jafari et al., 2018).

### 2.3 Shrinkage

Shrinkage was assessed based on the changes in the sample volume which was measured gravimetrically by the displacement of toluene in a pycnometer (Roueita et al., 2020).

### 2.4 Rehydration ratio

The dried banana (10 ± 0.1 g) was added to distilled water (solid to liquid ratio of 1:40) and kept at ambient temperature for 16 h. The rehydration ratio (RR) of the samples was calculated based on the following equation (Noshad et al., 2012; Vega‐Gálvez et al., 2012):

$$RR\;=\;\frac{W_{r}\;‐W_{d}}{W_{d}}$$

where W_r_ is the dried sample weight, and W_d_ denotes the weight of the rehydrated sample.

### 2.5 Color

A Minolta colorimeter CR‐400 (Konica Minolta, Inc) was used to assess the color of the samples based on the CIELab color space. Total color difference (∆E) was used to examine the color changes in the samples (Jafari et al., 2018).

### 2.6 Total phenolic content

To investigate the total phenolic content (TPC) of the samples, 30 μl of the banana extract and 2.5 ml of 10% Folin–Ciocalteu were mixed. Then, 2.37 ml of deionized water plus 2 ml of 20% sodium carbonate were incorporated into the solution. The samples were kept in the dark for 90 min. Next, the optical density (OD) was determined at 760 nm using a spectrophotometer (WPA UV 1101, Biotech Photometer). TPC was expressed as mg/ml (Alizadeh Behbahani et al., 2020; Hatamian et al., 2020; Vázquez‐González et al., 2020).

### 2.7 Antioxidant activity

To evaluate the antioxidant activity, 0.1 ml of the banana extract was mixed with 2.9 ml of 0.1 mM DPPH in ethanol, and the solution was kept in darkness at ambient temperature for 30 min. Subsequently, the absorbance value was measured at 515 nm. Total antioxidant activity was expressed as the percentage of inhibition using the following equation (Alizadeh Behbahani et al., 2021; Alizadeh Behbahani & Shahidi, 2019; Hatamian et al., 2020):

$$\mathrm{Inhibition}\;\left(\%\right)\;=\left[\frac{\left(A515\;blank\;‐\;A515\;sample\right)}{A515\;blank}\right]\;\times \;100$$

### 2.8 Scanning electron microscopy

The microstructure of the samples was verified using a scanning electron microscope (LEO model 1455 VP, LEO Electron Microscopy Ltd) at 30 kV. After cutting the samples along the perpendicular axis, they were sputter‐coated with gold (Sputter Coater Model SC 7620, Quorum Technologies Ltd) that had been connected to a double‐sided sticky tape. The images were captured at 500× magnification.

### 2.9 Drying kinetics

To investigate the effects of the aerosolized citric acid and RF pretreatment on the drying kinetics of banana, the samples were dried at 50°C. The moisture ratio (MR) of the samples during drying was calculated using the following equation:

$$MR\;=\;\frac{M\;‐\;M_{e}}{M_{0}\;‐\;M_{e}}$$

where, M, M_0_, and M_e_ respectively represent the moisture content at any time, initial moisture content, and equilibrium moisture content (kg water/kg dry matter). The MRs were fitted to the equations presented in Table 1 using MATLAB (R2020a, Mathworks, Inc), and *R* ^2^, RMSE, and *χ* ^2^ were evaluated to select the best model for drying the different samples, which should have the maximum *R* ^2^ and the minimum RMSE and *χ* ^2^ (Forouzanfar et al., 2020).

**TABLE 1:** Mathematical models of drying presented by various authors

Model name	Model equation
Newton	MR = exp(‐a t)
Page	MR = exp(‐a t^c^)
Henderson and pabis	MR = a exp(‐k t)
Logarithmic	MR = a exp(‐k t) + c
Wang and Singh	MR = 1 + at + bt^2^
Diffusion approach	MR = a exp(‐k t) + (1‐a)exp(‐k b t)

Note

a, b, and c indicate model's parameters, dimensionless.

### 2.10 Statistical analysis

Analysis of variance (ANOVA) and Duncan's multiple range test were conducted using SPSS 23.0 (SPSS Science) to investigate the differences between the treatments. The experiments were at least triplicated, and the average of the replications was reported.

## 3 RESULTS AND DISCUSSION

### 3.1 Total phenolic content and antioxidant activity

The results of the impacts of RF intensity and exposure duration on the TPC of the dried banana are summarized in Table 2, whereby raising the intensity of RF increased the TPC of the samples, while it decreased with a rise in the RF exposure duration. The highest TPC was associated with the sample treated with a frequency of 40 MHz for 40 min, and the lowest TPC was related to the one treated with a frequency of 13.56 MHz for 80 min (Table 2). The RF pretreatment reduced the drying time, and hence the product was exposed to hot air for a shorter time; as a result, the degradation of TPC, caused by the contact with hot air during drying, was reduced. Consequently, the TPC of the dried banana was also reduced (Noshad & Ghasemi, 2020). Moreover, with increasing the RF exposure duration, TPC decreased in all the samples, which is probably due to further degradation of the phenolic compounds. Owing to the relationship between TPC and antioxidant activity, the amount of the antioxidant activity decreased with an increase in the RF exposure duration.

**TABLE 2:** Effects of RF intensity and exposure duration on TPC and antioxidant activity

Antioxidant activity (%)	TPC (mg/g)	Treatment
18.9 ± 0.4^a^	127.5 ± 9.2^e^	RF:13.56 MHz, 40 min
13.4 ± 0.2^b^	120.5 ± 9.1^e^	RF: 13.56 MHz, 80 min
9.2 ± 0.7^de^	252.1 ± 4.24^d^	RF:27.12 MHz, 40 min
11.3 ± 0.6^f^	155.1 ± 4.1^c^	RF: 27.12 MHz, 80 min
12.3 ± 0.2^ef^	307.3 ± 4.9^a^	RF:40 MHz, 40 min
10.5 ± 0.5^bc^	288.5 ± 7.7^ab^	RF: 40 MHz, 80 min

Note

Similar letters in each column indicate no significant difference between the data at (*p* > .05).

### 3.2 Color

The results of the impacts of the RF intensity and exposure duration on the color change in the dried banana are presented in Table 3. According to the results, increasing the RF intensity elevated the color changes in the samples, while increasing the duration of the RF exposure decreased the color changes in the samples. The highest color change value belonged to the sample treated with a frequency of 40 MHz for 40 min, while the lowest color change value pertained to the one treated with a frequency of 13.56 MHz for 80 min (Table 3) (Jiang et al., 2019; Zhang et al., 2020). As the duration of the RF exposure increased, the extent of enzymatic browning decreased in the samples because they were exposed to aerosolized citric acid for a longer time. This reduced the amount of color changes in the samples (Moon et al., 2020).

**TABLE 3:** Effects of RF intensity and exposure duration on color, firmness, rehydration ratio, and shrinkage of dried samples

Shrinkage (%)	Rehydration	Firmness (*N*)	Color (ΔE)	Treatment
54.7 ± 2.2^b^	2.28 ± 0.01^ab^	32.4 ± 8.5^ab^	33.2 ± 2.9^cd^	RF:13.56 MHz, 40 min
62.1 ± 0.8^ab^	2.34 ± 0.01^a^	12.3 ± 0.8^d^	23.9 ± 0.6^e^	RF: 13.56 MHz, 80 min
59.9 ± 4.9^ab^	2.1 ± 0.02^bc^	31.7 ± 4.7^ab^	38.5 ± 5.2^abc^	RF:27.12 MHz, 40 min
64.5 ± 0.7^ab^	2.28 ± 0.06^ab^	11.5 ± 2.4^d^	33.4 ± 0.8^cd^	RF: 27.12 MHz, 80 min
60.1 ± 2.8^ab^	2.15 ± 0.2^abc^	23.9 ± 0.7^cd^	39.9 ± 3.7^ab^	RF:40 MHz, 40 min
69.1 ± 1.4^a^	2.3 ± 0.1^ab^	20.7 ± 4.4^bc^	34.9 ± 0.9^bcd^	RF: 40 MHz, 80 min

Note

Similar letters in each column indicate no significant difference between the data at (*p* > .05).

### 3.3 Texture

According to the results (Table 3), the use of the RF pretreatment reduced the firmness of the dried samples. It damaged the cell wall and influenced the firmness and flexibility of the sample final tissue. The firmness of the samples decreased with increases in the RF intensity and exposure duration, which could be due to the damage in the structural components of the cell wall such as cellulose and pectin. This was caused by the use of RF and aerosolization in the sample tissue. Thus, it softened the tissue (Jiang et al., 2020; Zhang et al., 2020).

### 3.4 Rehydration ratio

Rehydration is employed to assess the extent of the damage caused by treatments in the drying or pre‐drying processes. RR is significantly influenced by the textural properties of a product, and low RR denotes the collapse of its internal structures (Noshad et al., 2012). According to the results (Table 3), increasing the RF intensity reduced the RR of the samples, while raising the duration of the RF exposure increased this response. In general, the RR of foodstuffs depends on the degree of the damage to the cell structure (Gong et al., 2020; Zhou et al., 2019).

### 3.5 Shrinkage

The results of the effects of the RF intensity and exposure duration on the shrinkage of the dried banana are displayed in Table 3. As can be seen, the use of the RF pretreatment had a significant effect on the increase in the shrinkage of the samples, so that the highest shrinkage was related to the sample treated with a frequency of 40 MHz for 80 min, and the lowest shrinkage was related to the one treated with a frequency of 13.56 MHz for 40 min (Table 3). A rise in the RF intensity and exposure duration raised the cell wall collapse and damage; an effect that caused the blockage of some microchannels in the tissue. As a result, shrinkage increased in response to the tensile stresses within the cell structure (Gong et al., 2020; Zhou et al., 2019).

The physicochemical properties of the samples treated with a frequency of 27.12 Hz for 40 min, the ones treated with 1% aerosolized citric acid for 40 min, and the ones with no pretreatment, dried at 50°C were compared to examine the impacts of the aerosolized citric acid on the qualitative properties of the dried banana. According to the results (Table 4), the aerosolized citric acid preserved the phenolic compounds of the samples to a higher extent, and the TPC decreased from 311 ± 3.4 mg/g in the fresh bananas to 252.1 ± 4.24 mg/g in the samples treated with a RF of 27.12 Hz for 40 min, 280.5 ± 8.1 mg/g in the ones treated with 1% aerosolized citric acid for 40 min, and 162.5 ± 10.8 mg/g in the ones with no pretreatment. The larger amounts of phenolic compounds in the samples with the aerosolized citric acid pretreatment were presumably due to the antienzymatic characteristic of citric acid, which inactivated enzymes such as polyphenol oxidase and peroxidase in the samples. Smaller amounts of phenolic compounds were decomposed in the samples, due to the inactivation of polyphenol oxidase, which led to the preservation of more phenolic compounds. The results in Table 4 denote that the color changes in the pretreated samples were fewer than those in the control, which was due to the reduction in the enzymatic and nonenzymatic browning reactions in the pretreated samples. According to the results (Table 4), the shrinkage and RR of the samples with pretreatment were significantly different (*p* < .05) from those of the control, as the samples with pretreatment had the lowest shrinkage and the highest RR, compared with the control. This difference indicated a better product quality in drying and less damage to the sample tissue.

**TABLE 4:** Physicochemical properties of samples dried at 50°C and pretreated with a RF of 13.56 Hz for 40 min, 1% aerosolized citric acid for 40 min, and no pretreatment

Rehydration	Shrinkage (%)	Color (∆E)	TPC	Time (min)	Aerosolization citric acid (%)	RF (MHz)
2 ± 0.05^b^	75.1 ± 3.2^a^	42.2 ± 1.8^a^	162.5 ± 10.8^c^	0	0	0
2.25 ± 0.01^a^	51.5 ± 4.7^b^	31.9 ± 2.1^b^	280.5 ± 8.1^a^	40	1	0
2.1 ± 0.07^b^	58.5 ± 2.9^b^	33.5 ± 1.3^b^	252.1 ± 4.24^b^	40	1	27.12

Note

Similar letters in each column indicate no significant difference between the data at (*p* > .05).

### 3.6 Scanning electron microscopy

**FIGURE 2:**
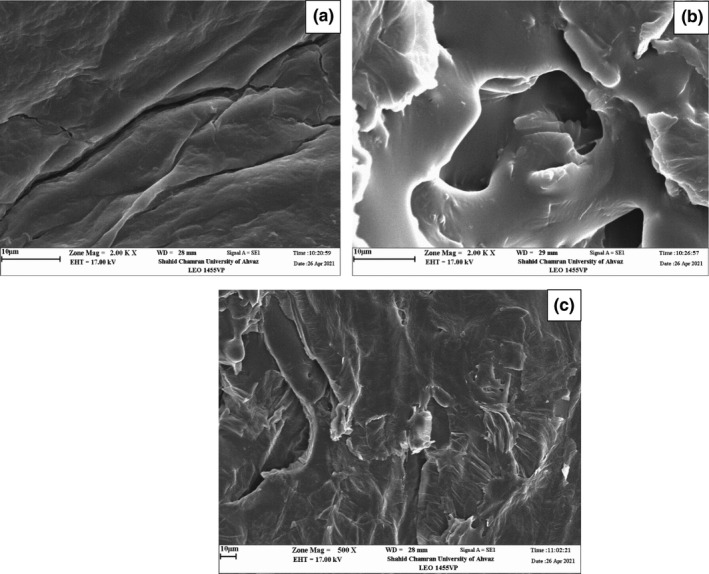
Micrographs of control (a), sample treated with aerosolized citric acid (b), and sample treated with aerosolized citric acid‐RF (c)

Figure 2 displays the impacts of RF pretreatment and aerosolized citric acid on the microstructure of banana. As can be seen, the application of the aerosolized citric acid pretreatment caused the tissue to soften and the formation of cell holes in the samples. Cell wall collapse and damage were more pronounced when RF was in use, which brought about the blockage of some microchannels in the tissue. In any case, the samples with pretreatment had more porous structures than the control.

### 3.7 Drying kinetics

**FIGURE 3:**
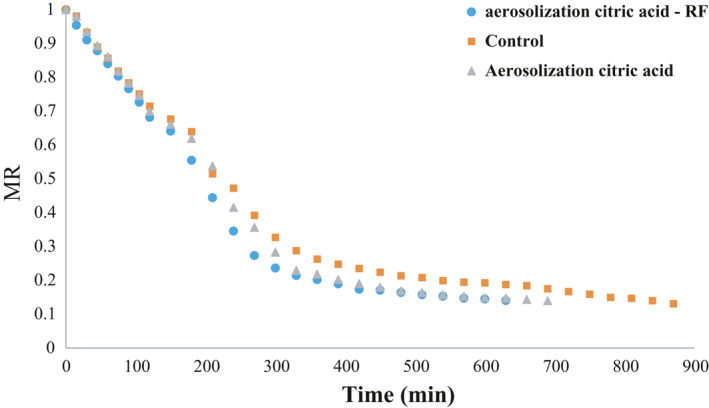
Effects of aerosolized citric acid and RF pretreatment on moisture ratio

The results indicated that the use of aerosolized citric acid and RF pretreatment reduced the drying time of banana (Figure 3) and eliminated the cell membrane resistance. Therefore, moisture could be transferred more easily from the cell interior to its exterior. As a result, moisture elimination from the product continued, and the drying time was reduced. In the control, the fine microchannels on the surface of the product were almost blocked, due to the elimination of moisture from the surface and shrinkage, and the blockage developed with further moisture elimination. Accordingly, such blockages reduced the moisture release and increased the drying time of the samples. As temperature rose, the drying time decreased, while the decomposition value of the membrane layers was elevated, the product tissue suffered more damage, and as a result, more moisture was released. Altogether, increasing the temperature intensified the molecular motion causing more water molecules to escape from the product and the drying time to be reduced (Forouzanfar et al., 2020; Noshad & Ghasemi, 2020). Different models were compared in terms of *R* ^2^, RMSE, and *χ* ^2^ to model the drying kinetics of the treated and control bananas. Finally, the model with the highest *R* ^2^ and the lowest RMSE and *χ* ^2^ was selected as the best model (Noshad & Ghasemi, 2020). Based on the results of Table 5, the Page model had the highest *R* ^2^ and the lowest RMSE and *χ* ^2^. Hence, it had the best fit with the experimental data at all the drying temperatures, the statistical results of which are given in Table 5.

**TABLE 5:** Statistical results and coefficients of models at different drying conditions

Model	Treatment	Constants	*R* ^2^	*χ* ^2^	RMSE
Diffusion approach	control	a = 0.41, k = 0.038, b = 0.18	0.963	0.038	0.041
	SA	a = 0.94, k = 0.05, b = ‐0.29	0.975	0.012	0.048
	RF + SA	a = 0.98, k = 0.012, b = ‐0.044	0.976	0.014	0.038
Logarithmic	control	a = 0.68, k = 0.005,c = 0.147	0.982	0.009	0.021
	SA	a = 0.86, k = 0.015, c = 0.187	0.978	0.017	0.078
	RF + SA	a = 0.92, k = 0.03, c = 0.088	0.981	0.008	0.024
Henderson and pabis	control	a = 0.71, k = 0.003	0.971	0.007	0.061
	SA	a = 0.81, k = 0.005	0.934	0.005	0.034
	RF + SA	a = 0.841, k = 0.005	0.979	0.006	0.028
Page	control	a = 0.036, c = 0.651	0.998	0.001	0.001
	SA	a = 0.031, c = 0.835	0.994	0.002	0.003
	RF + SA	a = 0.024, c = 0.928	0.996	0.001	0.002
Wang and Singh	control	a = ‐0.008,b = 0.00001	0.926	0.006	0.022
	SA	a = ‐0.009, b = 0.000017	0.958	0.006	0.018
	RF + SA	a = ‐0.006, b = 0.000014	0.971	0.002	0.017
Newton	control	a = 0.008	0.919	0.011	0.054
	SA	a = 0.0085	0.925	0.017	0.051
	RF + SA	a = 0.0094	0.957	0.008	0.046

Note

a, b, c, and k indicate model's parameters, dimensionless.

## 4 CONCLUSION

The application of aerosolized citric acid and RF pretreatment improved the quality of dried banana. Aerosolized citric acid and RF pretreatment also increased the TPC and antioxidant activity of the dried banana by decreasing the drying time. The Page model can be used to model the kinetics of drying. Therefore, aerosolized citric acid and RF pretreatment might be used by the industry as a pretreatment in banana drying to improve its quality.

## CONFLICT OF INTEREST

The authors have declared no conflict of interest.

## AUTHOR CONTRIBUTIONS

**Reza Ghorani:** Data curation (equal); Investigation (equal); Writing‐original draft (equal). **Mohammad Noshad:** Formal analysis (equal); Methodology (equal); Resources (equal); Software (equal); Supervision (equal); Writing‐original draft (equal); Writing‐review & editing (equal). **Behrooz Alizadeh :** Investigation (equal); Project administration (equal).

## ETHICAL APPROVAL

This article does not contain any studies with human or animal subjects.
